# Noninvasive high-frequency oscillatory ventilation versus bi-level positive pressure ventilation in premature infants with respiratory failure: A retrospective study

**DOI:** 10.12669/pjms.38.5.5939

**Published:** 2022

**Authors:** Wenqian Chen, Zhiqing Chen, Shuhua Lai, Wenhong Cai, Yunfeng Lin

**Affiliations:** 1 Wenqian Chen, Department of Neonatology, Fujian Maternity and Child Health Hospital, Affiliated Hospital of Fujian Medical University, Fuzhou City, China; 2 Zhiqing Chen, Department of Neonatology, Fujian Maternity and Child Health Hospital, Affiliated Hospital of Fujian Medical University, Fuzhou City, China; 3 Shuhua Lai, Department of Neonatology, Fujian Maternity and Child Health Hospital, Affiliated Hospital of Fujian Medical University, Fuzhou City, China; 4 Wenhong Cai, Department of Neonatology, Fujian Maternity and Child Health Hospital, Affiliated Hospital of Fujian Medical University, Fuzhou City, China; 5 Yunfeng Lin, Department of Neonatology, Fujian Maternity and Child Health Hospital, Affiliated Hospital of Fujian Medical University, Fuzhou City, China

**Keywords:** Noninvasive high-frequency oscillatory ventilation, Bi-level nasal continuous positive airway pressure, Preterm infants, Neonatal respiratory failure, Invasive mechanical ventilation

## Abstract

**Objectives:**

Noninvasive high-frequency oscillatory ventilation (nHFOV) is a novel respiratory support mode for premature infants. This retrospective study aimed to compare the effect of nHFOV and bi-level nasal continuous positive airway pressure (BiPAP) in premature infants with neonatal respiratory failure (NRF) as initial noninvasive ventilation (NIV) support mode.

**Methods:**

We retrospectively analyzed medical records of preterm infants admitted to the tertiary neonatal intensive care units (NICUs) of Fujian Maternal and Child Health Hospital from January 2019 to December 2020. Preterm infants with the gestational age of 25-34 weeks, diagnosed with NRF, used nHFOV or BiPAP as the initial respiratory support mode were analyzed. The rates of invasive mechanical ventilation (IMV) within the first seven days after birth and adverse outcomes were compared between the two groups.

**Results:**

Two hundred fifty-five preterm infants were analyzed (128 in nHFOV group,127 in BiPAP group). There was no significant difference in baseline characteristics between the two groups. Compared with the BiPAP group, the nHFOV group had significantly lower need for IMV within the first seven days after birth (18/128 vs. 33/127, p = 0.01) and PCO2 at 12 and 24 hours post-treatment (46.34±5.24mmHg vs. 51.18±4.83mmHg, P<0.01; 40.72±4.02mmHg vs. 42.50±3.86mmHg, P<0.01). The incidence of BPD, ROP, air leak syndromes, IVH≥ grade 3, PVL, NEC≥II stage, abdominal distension, and nasal trauma were similar between the two groups.

**Conclusion:**

nHFOV significantly reduced the need for IMV and improved the elimination of CO2 compared with BiPAP in preterm infants with NRF without increasing the incidence of adverse effects.

## INTRODUCTION

Neonatal respiratory failure (NRF) refers to pulmonary ventilation and/or ventilation dysfunction caused by various reasons, resulting in circulatory hypoxia and/or carbon dioxide retention syndrome. At present, the standard models of respiratory support used in NRF include invasive mechanical ventilation (IMV) and noninvasive ventilation (NIV). IMV can bring serious complications, such as bronchopulmonary dysplasia (BPD), pneumothorax, ventilator-associated pneumonia, and subglottic stenosis.^1^ In contrast, NIV can reduce the rate of tracheal intubation, BPD and reduce the total oxygen time.^2^ Therefore, noninvasive ventilation technology has gradually become the method of choice in the initial treatment of NRF.

US and European guidelines recommend early application of NIV to treat respiratory diseases in premature infants to obtain ideal results.^3^ Frequently used NIVs in neonatal intensive care units (NICUs) are nasal continuous positive airway pressure (nCPAP), nasal intermittent positive pressure ventilation (NIPPV), bi-level nCPAP (BiPAP), and humidified high flow nasal cannula (HHHFNC).^4^ However, clinical studies have found that for 38%-42% of very low birth weight infants, the traditional nCPAP treatment is not sufficient, and they require invasive ventilation.^5^

In recent years, nasal noninvasive high-frequency oscillatory ventilation (nHFOV), a relatively novel mode of noninvasive ventilation, has gradually been applied in many NICUs. This mode uses nasal prong, facemask, or nasopharyngeal tube to apply oscillating pressure waves to the lungs. By combining the advantages of noninvasive ventilation and high-frequency ventilation, nHFOV provides optimal respiratory support.^6^ At present, there are studies on treatment of neonatal respiratory distress syndrome with nHFOV and the prevention of post-extubation failure.^7^,^8^ However, there are only few reports of using nHFOV as the initial respiratory support mode to treat respiratory failure in premature infants.^9^

The primary objective of this study was to compare safety and efficacy of bi-level positive pressure ventilation (BiPAP) and noninvasive high-frequency oscillatory ventilation (nHFOV) as an initial respiratory support mode in premature infants with respiratory failure to find a safer and more effective noninvasive ventilation mode.

## METHODS

Medical records of 307 premature infants, admitted to the III-level neonatal intensive care unit (NICU) of Fujian Maternity and Child Health Hospital from January 2019 to December 2020, were retrospectively evaluated. The study was approved by the Ethics Committee of Fujian Provincial Maternal and Child Health Hospital (number 2021YJ009, date: 2021-04-27) and conducted according to the Good Clinical Practice Guideline and the Declaration of Helsinki.

### Inclusion criteria:

- Gestational age between 25 and 34 weeks;
- Patients with neonatal respiratory failure (as described below) diagnosed within one hour after birth and received nHFOV or Bipap assisted ventilation.
- The diagnostic criteria for neonatal respiratory failure were based on clinical manifestations and arterial blood gas analysis. The clinical signs and symptoms of NFR were as described in Reuter et al. respiratory distress, groan, breathing concave signs, cyanosis, and circulatory disorder.^10^ The arterial blood gas analysis showed arterial oxygen partial pressure (PaO2) < 60 mmHg and/or carbon dioxide partial pressure (PaCO2) > 50 mmHg at sea-level atmospheric pressure under resting conditions and breathing room air in the absence of a cardiac anatomic shunt and decreased primary cardiac output.^11^

### Exclusion criteria:

- Severe asphyxia (1 minute Apgar <4 at 1minutes, or cord blood pH <7.0);
- Cyanotic congenital heart disease (cCHD);
- Maxillofacial developmental abnormalities (cleft palate, distorted nasal septum, atresia of posterior foramen);
- Chromosomal, genetic metabolic disease;
- Shock;
- Give up treatment within seven days of admission.

After birth, normal body temperature of premature infants was maintained, and airway cleared. Preterm infants who have respiratory failure were given T-piece resuscitator (CPAP 5-8 cmH2O) support and transferred to the NICU within 1 hour after birth to receive nHFOV or Bipap.

The nHFOV was provided by VN 500 (Drager, Lubeck, Germany) or SLE 5000 (SLE, croydon, UK). The following parameters were settled:

- MAP: 6-12cmH0
- Frequency: 6-12 Hz;
- Amplitude: is two to three times that of MAP, based explicitly on visible oscillations of the chest.

The BiPAP was provided by SiPAP (CareFusion, California, US). BiPAP parameters setting were as follows: the lower positive pressure is 5-7cmH20; the higher positive pressure is 8-12cmH20, and the higher positive pressure setting is more than the lower positive pressure 3-5cmH20; the higher positive pressure is maintained for 0.5 to 0.7 seconds, and the ventilator frequency is 30 breaths/min.^12^

FiO2 was adjusted by a respiratory therapist to obtain a target SpO2. The target SpO2 was measured by preductal pulse oximetry ranges from 89% to 93% for preterm infants <30 weeks gestational age and from 90% to 94% for preterm infants ≥30 weeks gestational age.^13^ FIO2 should be no more than 60% to avoid potential damage at higher concentrations.

The primary outcome was the need for intubation and mechanical ventilation within the first seven days of life. The criteria for intubation and mechanical ventilation were severe respiratory acidosis (defined as pH < 7.20 with PaCO2> 65 mmHg); severe apnea and bradycardia (defined as recurrent apnea with more than three episodes per hour associated with heart rate less than 100 beats per minute or a single episode of apnea that requires mask ventilation) and hypoxia (defined as FiO2>0.5 with PaO2 < 50 mmHg) for more than two hours.^14^

Secondary outcomes included flowing aspects:pCO2 and PO2 levels(PO2 and PCO2 were obtained before and during the NIV treatment at several time points (12 and 24hrs after the start of NIV treatment), PFr (PFr = PO2/ FiO2), distribution of SpO2(0-24h after NIV), the incidence of mortality before discharge, traumatization of nasal skin and mucosa, abdominal distension, BPD (defined as the requirement for oxygen or positive pressure support at 36 weeks postmenstrual age or discharge home, whichever was sooner^15^), intraventricular hemorrhage (IVH) grade≥3 (according to the Papile classification^16^), periventricular leukomalacia (PVL), necrotizing enterocolitis (NEC), retinopathy of prematurity (ROP) and air leaks (including pneumothorax, pneumomediastinum, and pneumopericardium).

The transcutaneous oxygen saturation (SpO2) was continuously measured using BeneVision N12 (Mindray, Shenzhen, China) percutaneous oxygen saturation monitor that collected percutaneous oxygen saturation (SpO2) data every second. Percutaneous oxygen saturation data 0-80%, 81-85%, 86-90%, 91-95%, and 95-100%, were collected and automatically counted every 24 hours through the built-in algorithm, and the percentages of each group were displayed in a certain period.

SPSS 22.0 software was used for statistical analysis of the data. According to previous studies, the failure rates of nHFOV and BiPAP for respiratory support in preterm infants are approximately 14% and 34.0%, respectively.^17^ Set α=0.05 (two-sided), power=0.80, and the ratio of nHFOV sample size to BiPAP sample size were 1:1. Sample size calculation by PASS 11 indicated that at least 109 preterm infants would be needed in each group. The measurement data that conform to the normal distribution and homogeneity of variance were expressed as x±s. The comparisons between the two groups were performed by two independent sample ***t-test***. The measurement data that does not conform to the normal distribution were expressed as median and IQR (*M (IQR)*). The *Mann-Whitney U* test was performed for the comparisons between the two groups. The count data were expressed as a percentage and comparison between two groups using the ***Chi-square test*** or ***Fisher’s exact probability*** method. P < 0.05 was considered statistically significant.

## RESULTS

Study included 128 infants with nHFOV and 127 infants with BiPAP. There were no statistically significant differences in any of the baseline characteristics of maternal, perinatal, or neonatal variables (Table-I). The primary causes of neonatal respiratory failure were neonatal respiratory distress syndrome (NRDS), neonatal pneumonia, and the newborn’s transient tachypnea (TTN). TTN mainly manifested as shortness of breath soon after birth and subsided spontaneously within 2-5 days after onset, with typical clinical and imaging manifestations.^17^ There was also no significant difference in the primary disease composition ratio between the two groups.

**Table I:** Baseline characteristics.

	nHFOV (N=128)	BiPAP (N=127)	P-value
GA (weeks) mean (s.d.)	29.39±2.66	30.05±3.34	0.08
BW (g) mean (s.d.)	1410.95±500.37	1461.41±570.50	0.45
Male n (%)	83 (64.8%)	87 (68.5%)	0.53
Twin n (%)	27 (21.1%)	24 (15.9%)	0.66
Chorioamnionitis n (%)	67 (52.3%)	73 (57.6%)	0.41
Cesarean section n (%)	74(57.8%)	67(52.8%)	0.41
Apgar score at 1 min median (IQR)	8(8,9)	8 (7,9)	0.26
Apgar score at 5 min median (IQR)	10(9,10)	10(9,10)	0.07
PROM≥18h n (%)	22(17.2%)	26(20.5%)	0.50
NRDS n (%)	87(68%)	88(69.3%)	0.82
Pneumonia n (%)	34(26.5%)	30(23.6%)	0.58
TTN n (%)	7(5.5%)	9(7.1%)	0.59

***Abbreviations:*** nHFOV, Noninvasive High-Frequency Ventilation; BiPAP, Bi-Phasic Continuous Positive Airway Pressure; GA, Gestational Age; BW, Birth Weight; IQR, Interquartile Range; PROM, Premature Rupture of Membranes; NRDS, Neonatal Respiratory Distress Syndrome; TTN, Transient Tachypnea of the Newborn.

The rate of IMV in the nHFOV group was significantly lower than the BiPAP group within seven days after birth. The number of infants with severe respiratory acidosis and severe apnea requiring IMV was significantly lower in nHFOV group than in BiPAP group (P<0.05). In contrast, there was no difference between the two groups in the requirement for IMV due to hypoxemia (Table-II). The PCO2 of nHFOV group was significantly lower than of BiPAP one at 12 hours and 24 hours after NIV (Table-III). There was no significant difference in PFr between the two groups before and after treatment. Within 24 hours after nHFOV, the distribution of blood oxygen saturation in the range of 91%-95% was significantly higher than in infants in BiPAP group (83.84±6.43% vs. 79.21±8.60%, P<0.01). In contrast, the distribution of blood oxygen saturation in the range of 0%-85% and 96%-100% was not significantly different (Table-IV). There were no significant differences in the incidence of abdominal distension, nasal mucosal injury, air leak, NEC, ROP, BPD, IVH grade 3-4, PVL, and mortality between the two groups (Table-V).

**Table II:** Incidence and causes of intubation within the first seven days. A Fisher’s exact test.

	nHFOV (N=128)	BiPAP (N=127)	P-value
Incidence of Intubation≤7d n (%)	18(14.1%)	33(26.0%)	0.01
Severe Respiratory Acidosis n (%)	3(2.3%)	12(9.4%)	0.01
Severe Apnea And Bradycardia n (%)	2(1.6%)	9(7.1%)	0.03
Hypoxia n (%)	13 (10.2%)	12 (9.4%)	1.00

**Table III:** Changes in PaCO2 level before and after NIV.

PCO2(mmHg)(s.d)	nHFOV (N=128)	BiPAP (N=127)	P-value
Before NIV	56.54±6.00	55.62±5.18	0.19
12h after NIV	46.34±5.24	51.18±4.83	<0.01
24h after NIV	40.72±4.02	42.50±3.86	<0.01

***Abbreviations:*** PaCO2, partial pressures of carbon dioxide; NIV, noninvasive ventilation.

**Table IV:** Oxygenation within 24 hours after NIV.

	nHFOV (N=128)	BiPAP (N=127)	P-value
PFr before NIV(S.d)	164.42±35.99	167.95±35.26	0.43
PFr 12h after NIV(S.d)	211.34±28.81	208.35±27.09	0.39
PFr 24h after NIV(S.d)	237.47±27.55	233.92±28.69	0.31
**The proportion of SpO2first 24 hours %**			
0%-80% (S.d)	0.49±0.71	0.54±0.58	0.53
81%-85% (S.d)	0.80±0.63	0.83±0.89	0.75
86%-90% (S.d)	5.50±3.76	9.63±4.86	<0.01
91%-95% (S.d)	83.84±6.43	79.21±8.60	<0.01
96%-100% (S.d)	9.37±4.57	9.79±4.39	0.45
0%-90% (S.d)	6.79±3.96	11.00±4.96	<0.01
91%-100% (S.d)	93.21±3.96	89.00±4.93	<0.01

***Abbreviations:*** PFr, PO2 to FiO2 ratio, PFr = PO2/ FiO2; SpO2, percutaneous oxygen saturation; NIV, Noninvasive Ventilation.

**Table V:** Comparison of outcomes in nHFOV and BiPAP groups.

	nHFOV (N=128)	BiPAP (N=127)	P-value
TNKM n(%)	43(33.6%)	42(33.1%)	0.92
AD n(%)	23(18%)	33(26%)	0.12
Air Leaks n(%)	0 (0)	2 (1.6%)	0.15
NEC n(%)	5(3.9%)	10(7.9)	0.17
ROP n(%)	10(7.8%)	11(8.7%)	0.80
BPD n(%)	21(16.4%)	26(20.5%)	0.40
IVH≥ grade 3 n(%)	5(3.9%)	6(4.7%)	0.74
PVL n(%)	4(3.1%)	4(3.1%)	1.00^a^
Death n(%)	1(0.8%)	3(2.4%)	0.37^a^

***Abbreviations:*** TNKM, Traumatization of Nasal Skin and Mucosa; AD, Abdominal Distension; NEC, Necrotizing Enterocolitis; ROP, Retinopathy of Prematurity; BPD, Bronchopulmonary Dysplasia; IVH, Intraventricular Hemorrhage; PVL, Periventricular Leukomalacia; A Fisher’s exact test.

## DISCUSSION

Our study showed that nHFOV can significantly reduce the need for IMV and is associated with the improved elimination of CO2 in preterm infants with NRF, without increasing the rates of adverse effects.

Noninvasive high-frequency oscillatory ventilation (nHFOV) is a novel noninvasive ventilation mode that uses airflow through nasal congestion or a nasal mask to generate continuous positive pressure. nHFOV combines the advantages of nCPAP and high-frequency ventilation, superimposes on this pressure with high-frequency oscillations that exceed physiological ventilation and achieve effective gas exchange. The main advantage of nHFOV is that, while being non-invasive, it can quickly improve oxygenation and eliminate carbon dioxide while maintaining continuous lung expansion and low tidal volume.

The use of nHFOV has previously been evaluated in animal models and has shown promising results. Mukerji et al.^18^ compared nHFOV with NIPPV and CPAP in an in-vitro lung model with standard lung mechanics and demonstrated that nHFOV has excellent CO2 elimination ability. Compared with IMV, nHFOV can maintain oxygenation and ventilation and improve alveolar/lung development^19^, significantly reducing CO2 retention and the onset of apnea.^20^ Clinical retrospective studies have shown that the overall effective rate of nHFOV in very low birth weight infants was 89%.

Studies show that intubation can be avoided in 88% of patients by switching to nHFOV. However, there is still no consensus on whether nHFOV can reduce the tracheal intubation rate. Zhu et al.^21^ showed that nHFOV could significantly reduce the rate of tracheal intubation compared with nCPAP. On the other hand, Mukerji et al.^22^ showed that in premature infants weighing less than 1250g and failed CPAP treatment, the tracheal intubation rate was slightly but not significantly lower in the nHFOV group as compared to BiPAP group. In our study, the rate of tracheal intubation in the nHFOV group was significantly lower than that in the BiPAP group. Our results are in agreement with the results of a recent meta-analysis on the application of nHFOV in respiratory support in preterm infants that showed that nHFOV can significantly reduce tracheal intubation rate compared with nCPAP and Bipap.^23^

Premature babies are more prone to apnea due to collapse of the chest wall, low diaphragm strength, and the glottis being closed during the inhalation phase. Compared with nCPAP, nHFOV does not induce the glottis contraction muscles’ activity, thereby obtaining sufficient gas exchange and reducing apnea risk.^24^ Our study found that the rate of severe apnea that requires tracheal intubation in the nHFOV group was significantly less than that of BiPAP. The results of studies such as Mukerji^6^ also confirmed that the use of nHFOV in newborns could reduce the number of apneas, PCO2, or heart rate drops.

Our study found that number of patients with severe hypercapnia requiring tracheal intubation were significantly lower in the nHFOV group than in the BiPAP group. The PCO2 level of the two groups showed no significant differences before NIV. However, at 12 hours and 24 hours after NIV, the nHFOV group had a lower PCO2 level than the BiPAP group. Our research shows that nHFOV can improve lung ventilation faster than BiPAP and have better CO2 clearance, which is consistent with the results of Roberto Bottino et al.^25^ However, Klotz et al.^26^ reported that, compared with nCPAP, nHFOV in premature infants could not increase CO2 clearance. This apparent difference in outcomes may be related to the lower PCO2 of the included subjects.

The increase in PFr after NIV was similar in both groups, suggesting that both ventilation modes can improve oxygenation. The distribution of SpO2 within 24 hours after NIV showed no difference in the domain of SpO2 lower than 0%-85%. However, the SpO2 of the BiPAP group was more in the 85%-90% range, while the SpO2 of nHFOV group was more in the 91-95% range, indicating that nHFOV oxygenation is better than BiPAP. Additionally, there was no significant difference in the 95%-100% interval, indicating that nHFOV is not associated with the increased hyperoxia damage. It is possible that nHFOV provides continuous positive pressure, the average airway pressure is relatively stable, and nHFOV does not need to synchronize with spontaneous breathing. Compared with BiPAP, nHFOV can provide more stable and adequate ventilation, reducing apnea and making blood oxygen saturation more stable. Adequate oxygenation further reduces the occurrence of central apnea and forms a virtuous cycle in which adequate oxygenation reduces the occurrence of apnea, and the reduction of apnea is conducive to the stability of blood oxygen saturation.

The low tidal volume used in the nHFOV mode and the optimal pressure that can maintain the continuous expansion of the alveoli can minimize the damage caused by volume injury and alveolar collapse, thereby reducing the occurrence of ventilator-related lung injury.^27^ Our study showed that there was no significant difference between both groups in the rate of air leakage, BPD, nasal mucosal injury, abdominal distension, NEC, ROP, IVH, PLV, and mortality. These results indicated that nHFOV would not increase adverse reactions.

### Limitations of the study:

This is a retrospective study with a relatively small sample size. Additionally, since the initial parameter settings of nHFOV and BiPAP may be different, the difference in results may be caused by any difference in parameter settings rather than the waveforms generated by the two noninvasive ventilation modes. There is a need for high-quality research to develop recommendations for the ideal combination of nHFOV settings that can exert the best ventilation effect. Moreover, the long-term safety and long-term neurological effects of nHFOV use are still unknown, requiring further research.

## CONCLUSION

Our study shows that nHFOV is more effective than BiPAP in promoting the discharge of CO2 and reducing the rate of tracheal intubation without increasing the incidence of air leakage, BPD, NEC, ROP, IVH, and other adverse reactions. It can be used as the initial stage of respiratory failure in premature infants.

### Authors’ Contributions:

**WC:** Conceived and designed the study.

**ZC, SL, WC & YL:** Collected the data and performed the analysis.

**WC:** Was involved in the writing of the manuscript and is responsible for the integrity of the study.

All authors have read and approved the final manuscript.
